# Active coping strategies and less pre-pandemic alcohol use relate to college student mental health during the COVID-19 pandemic

**DOI:** 10.3389/fpsyg.2022.926697

**Published:** 2022-08-01

**Authors:** Elisabeth Akeman, Mallory J. Cannon, Namik Kirlic, Kelly T. Cosgrove, Danielle C. DeVille, Timothy J. McDermott, Evan J. White, Zsofia P. Cohen, K. L. Forthman, Martin P. Paulus, Robin L. Aupperle

**Affiliations:** ^1^Laureate Institute for Brain Research, Tulsa, OK, United States; ^2^School of Community Medicine, The University of Tulsa, Tulsa, OK, United States; ^3^Department of Psychology, The University of Tulsa, Tulsa, OK, United States; ^4^Department of Psychology, Oklahoma State University, Stillwater, OK, United States

**Keywords:** college, depression, anxiety, COVID-19, resilience

## Abstract

**Objective:**

To further delineate risk and resilience factors contributing to trajectories of mental health symptoms experienced by college students through the pandemic.

**Participants:**

*n* = 183 college students (67.2% female).

**Methods:**

Linear mixed models examined time effects on depression and anxiety. Propensity-matched subgroups exhibiting “increased” versus “low and stable” depression symptoms from before to after the pandemic-onset were compared on pre-pandemic demographic and psychological factors and COVID-related experiences and coping strategies.

**Results:**

Students experienced worsening of mental health symptoms throughout the pandemic, particularly during Fall 2020 compared with Fall 2019 (Depression scale *d* = −0.43 [95% CI: −0.65 to −0.21]). The propensity-matched subgroup exhibiting relative resilience (“low and stable” symptoms) reported less alcohol use prior to the pandemic, greater use of active coping strategies, and less of an impact on their college progress.

**Conclusions:**

Results point to several potential targets of screening and intervention to decrease residual impacts of the pandemic.

## Introduction

On January 30, 2020, the World Health Organization declared the COVID-19 (i.e., SARS-CoV-2) outbreak a public health emergency of international concern (World Health Organization, 2020). In March and April 2020, the United States began implementing safety protocols to limit the spread of the virus. Safety provisions included mask mandates, social distancing measures, and lockdowns (Centers for Disease Control and Prevention, 2020). For many, this marked a dramatic life change, cutting people off from friends and family, introducing increased health and financial concerns, and restricted everyday activities. The global influence of COVID-19 also provides an opportunity to examine the vulnerability and resilience factors moderating the impact of a severe life event. Delineating pre-existing and concurrent psychological, behavioral, and environmental factors that increased risk for or protect against negative mental health outcomes during the COVID-19 pandemic will be useful for informing how we may optimize responses to future negative world events and enhance human resilience in general.

Previous research indicates that the COVID-19 pandemic has had detrimental effects on the psychological and emotional health of the general population, contributing to increased depression, anxiety, and loneliness (Marroquín et al., 2020; Tull et al., 2020). However, some studies have reported no change or even a decline in mental health symptoms (e.g., decreased suicide rates) (Pirkis et al., 2021). There is some evidence to suggest that the COVID-19 pandemic and related social distancing measures may have had a particularly negative impact on the mental health of young adults, including college students specifically (Kecojevic et al., 2020; Son et al., 2020). Previous research has highlighted that college is a significant stressor and that college students are at greater risk for developing mental health disorders than the general population (Hunt and Eisenberg, 2010). Pre-pandemic, the 1-year prevalence rate of anxiety and depressive disorders in college students was estimated between 15 and 30% (compared with rates between 7 and 18% for the general adult population) (Kessler et al., 2005; Hunt and Eisenberg, 2010; Ibrahim et al., 2013). Concerns about college student mental health have only been strengthened with the onset of the COVID-19 pandemic, as over 70% of college students report increased stress and anxiety because of the pandemic and an estimated 48% experience moderate to severe levels of depression after the onset of the pandemic (Hunt and Eisenberg, 2010; Son et al., 2020; Wang X. et al., 2020).

There are numerous factors related to the COVID-19 pandemic response that have likely impacted college student mental health. For example, students have faced not only concerns about the virus itself and social isolation but also disruptions in academic progress toward graduation, sudden changes in the structure of coursework (i.e., to virtual format), financial hardships, and decreased job opportunities (Lederer et al., 2020; Hawley et al., 2021). Previous research has reported that students’ greatest concerns during the pandemic include worry for their health and that of their families, disruption of sleep patterns, difficulty concentrating, and increased concern for academic performance (Son et al., 2020).

Prior studies have also identified coping strategies that individuals have found helpful in managing their wellbeing during the COVID-19 pandemic. Research suggests that approach-based coping strategies, positive reframing, access to social support, and findings ways to stay connected with friends and family, healthy lifestyle activities (e.g., exercise, sleep, healthy eating, self-care), engagement in faith-based activities, and access to and use of greenspace may be beneficial (Wang S. et al., 2020; Cohen et al., 2021; Shamblaw et al., 2021; Soga et al., 2021). On the other hand, maladaptive coping strategies such as distraction techniques, excessive alcohol use, denial, and isolation, have been associated with lower levels of mental health and quality of life in response to the COVID-19 pandemic (Wang X. et al., 2020; Shamblaw et al., 2021).

Studies have identified numerous pre-pandemic risk factors that may impact a person’s likelihood of developing mental health symptoms in response to the pandemic. These include having a history of mental health symptoms, being single or divorced, lower education level, frequent exposure to COVID-related news, or identifying as a racial minority or LGBTQ+ (Gonzales et al., 2020; Xiong et al., 2020; Fruehwirth et al., 2021). Researchers have also identified potential resilience factors, that is, potential reasons that one may be able to better adapt and cope with the pandemic including optimism, religiosity or faith, greater levels of social support, approach-based coping strategies (Lawal et al., 2020; Pirutinsky et al., 2020; Wang S. et al., 2020; Vos et al., 2021).

There have only been a few studies reporting changes in mental health from before to after the pandemic for the same population of students (Copeland et al., 2021). The current study sought to extend previous work by (1) examining the trajectory of depression and anxiety symptoms for a cohort of college students who were followed from 1 year before to approximately 6 months after the COVID-19 pandemic began and (2) identify factors contributing to different trajectories of mental health response. For the latter, groups of students matched on sociodemographic variables and pre-pandemic mental health symptoms who showed different trajectories of response to the COVID-19 pandemic were identified to enable examination of (1) pre-pandemic external/environmental and internal/psychological factors and (2) COVID-related experiences and coping strategies, that contributed to mental health trajectory.

## Materials and methods

### Participants

Participants in this study were students from a private, mid-Western university who voluntarily enrolled in a larger, longitudinal study designed to increase resilience in university students during their first year (Akeman et al., 2019). Participants were recruited during the first semester of their enrollment at the college (Fall semester, cohorts recruited in 2016, 2017, and 2018) as part of a previously conducted clinical trial. Participants were asked to complete demographic and self-report measures at the beginning of their first semester of college and once per semester thereafter for the following 5 years. The current analysis focused on a sample of 177 students (67.8% female) who completed surveys during at least one of the three timepoints prior to the start of the COVID-19 pandemic (Spring 2019, Summer 2019, and Fall 2019), and the timepoint after the start of the COVID-19 pandemic, in which we added surveys specifically related to their experiences related to COVID-19 (Summer 2020). Included in analysis were also timepoints corresponding to the semester in which the pandemic began but no COVID-19 related surveys were implemented yet (Spring 2020) and the Fall 2020 timepoint in which all surveys (including COVID-19 specific surveys) were repeated. Thus, in total, we included data from three time points prior to the beginning of the COVID-19 pandemic and three timepoints at or after the start of the pandemic. A timeline of state and local government restrictions and trajectory of total cases in the region in relation to study survey time points is provided in Supplementary Figure 1. The participants included in the current study overlap with the participants included in a previous study examining clinical outcomes of a resilience-based intervention for first-year college students (Akeman et al., 2019).

Participants were excluded if they were under 18 years of age, not in their first year of college, unable to understand the consent form or surveys presented in English, or if they reported significant mental or physical health problems requiring immediate medical attention. In accordance with federal and university regulations preventing students on international visas from receiving research compensation, these students were also excluded. All students provided written informed consent prior to participation and were compensated for their time. Research was approved by the Western Institutional Review Board and conducted in accordance with the World Medical Association Declaration of Helsinki. The study was registered at the United States National Institutes of Health (ClinicalTrials.gov #NCT02982070).

### Measures

All measures were completed via secure survey links through Research Electronic Data Capture (REDCap) (Harris et al., 2009). This survey capture method is designed in a way that all fields must be completed before submission, thereby eliminating missing questions within surveys. Demographic surveys obtained information related to gender, race, ethnicity, current college within the university (Arts and Sciences, Business, etc.), parent/household income, financial aid amount received for college, whether they were the first in their family to attend college, and whether they had received psychological treatment. The primary outcome measure was the National Institute of Health Patient Reported Outcome Measurement Information System (PROMIS) computer adaptive Depression symptom measure, with the PROMIS Anxiety symptom measure serving as a secondary outcome (Cella et al., 2010; Gershon et al., 2010). PROMIS Depression was selected as the primary outcome based on previous literature which highlighted the prevalence of depression with the COVID-19 pandemic (Ettman et al., 2020; Wang X. et al., 2020; Bueno-Notivol et al., 2021). Other measures of interest for assessing pre-pandemic psychological risk and resilience included PROMIS measures for sleep impairment, sleep disturbance, social isolation, emotional support, and informational support (Cella et al., 2010; Gershon et al., 2010); NIH Toolbox measures for meaning and purpose, positive affect, friendship, self-efficacy, and perceived stress (Salsman et al., 2013a,b, 2014), the Connor-Davidson Resilience Scale (CD-RISC 10) total score (Connor and Davidson, 2003); the Emotion Regulation Questionnaire (ERQ) total, reappraisal, and suppression subscale scores (Gross and John, 2003); Alcohol, Smoking and Substance Involvement Screening Test (Group, 2002) for assessing alcohol and cannabis use (Group, 2002), the Epworth Sleepiness Scale (ESS) (Johns, 1992); and an item asking students to rate how important religion is to them on a 1–7 Likert scale.

For Summer and Fall 2020 time points (i.e., after pandemic onset), participants also completed surveys consisting of (1) aspects of the COVID-19 Adolescent Symptom & Psychological Experience Questionnaire [CASPE (Ladouceur, 2020)] and (2) the COVID Wellbeing scales (Veldhuis et al., 2021). For current analysis, we focused on (1) the Brief Cope Scale, which asked students to rate how often they utilize different coping skills “right now” and results in subscales for acceptance, distraction, active coping, denial, substance use, emotional support, instrumental support, behavioral disengagement, venting, positive reframing, planning, faith or religion, humor, and self-blame, (2) COVID Wellbeing scale in which students indicated how much they are worried “right now” about the following aspects of the COVID-19 outbreak (rated on a scale of 0–100): the coronavirus, their own health, their family’s health, money, their job, their future, and their performance at college (added specifically for this study), and (3) a scale in which participants rated how much time they were spending on the following activities each day: school work, social media, video games, reading books, talking to friends and family, engaging in fun activities, work, exercising, watching/reading the news, or watching tv/movies. Additional variables of interest included whether the participant, family member, friend, or anyone they knew had been diagnosed or were hospitalized with COVID-19, as well as whether anyone in their family had died due to COVID-19, the level of engagement in social distancing, and one’s political views (rated 1–8, with 1 = extremely liberal and 8 = extremely conservative). This latter variable was included because political views have the potential to relate to one’s experience and opinions concerning the COVID-19 pandemic and given that the COVID-19 pandemic was overlaid upon a relatively tumultuous political context in the United States (Bruine de Bruin et al., 2020; Calvillo et al., 2020).

### Statistical analysis

Statistical analyses were conducted using R 4.0.4 (R Core Team, 2021). Linear mixed models (LMM); conducted by “lme4” package (Bates et al., 2015), with subject entered as a random effect, were used to determine whether there were time effects on depression (primary outcome) and anxiety (secondary outcome) symptoms. The inclusion of potential covariates (gender; college; cohort) were determined by comparing models using the Bayesian Information Criterion (Bicanic et al., 2015). The use of LMM allowed for the inclusion of participants who may have not completed some timepoints, while making use of the data that was available for each participant. Thresholds for significance for symptom outcomes was set to *p* < 0.05. Tukey’s HSD (honestly significant difference) tests were used to examine differences between paired time points from before to after the beginning of the pandemic (i.e., comparing Spring 2019 to Spring 2020; Summer 2019 to Summer 2020; and Fall 2019 to Fall 2020), with confidence intervals and effect sizes estimated using the “emmeans” package (Russell, 2021).

Subgroups of participants were identified concerning the profile of symptom response to the COVID-19 pandemic. Specifically, we identified three groups of students (1) Increased depression: those whose average PROMIS Depression scale after the start of the pandemic was at least 3.5 points higher than the average before the start of the pandemic, (2) High stable depression: those with less than 3.5 points increase on PROMIS Depression (or a decrease in symptoms) but with relatively high pre-pandemic symptoms (>55 T score, averaged across pre-pandemic time points), and (3) Low stable depression: those with less than 3.5 points increase on PROMIS Depression (or a decrease in symptoms) but with relatively low pre-pandemic symptoms (<55 T score, averaged across pre-pandemic time points). The cutoff of 3.5 T points for change in symptoms and the cutoff of *T* = 55 for symptom severity was based on the minimally important difference (MID) and the cutoff associated with mild symptom severity identified in previous research on the PROMIS Depression scale (Kroenke et al., 2020). To support analysis identifying factors that may contribute to students’ mental health risk versus resilience with the COVID-19 pandemic, we focused on the “increased” and the “low stable” depression groups. Focusing on these two groups allowed for us to compare subgroups that had similar pre-pandemic symptom measures but for whom the mental health response to the pandemic differed (whereas we were unable to match pre-pandemic symptoms for the “high stable” group with the other groups). Using the “MatchIt” package (method = “optimal”; distance = “glm”) (Ho et al., 2011), we identified cases in the “low stable” group that matched the “increased” group on gender, race (binary variable: minority, white), ethnicity (binary: Hispanic vs. non-Hispanic), cohort (2016, 2017, 2018), and pre-pandemic average PROMIS Depression score. This resulted in a total sample of 632126 for analysis with the matched groups. We chose to use optimal pair matching, as it minimizes the sum of the absolute pairwise distances in the matched samples.

Mann-Whitney tests and chi-square analyses were used to compare these groups on the following COVID-related experiences and responses: (1) COVID-19 health experiences (i.e., having or knowing others with COVID, hospitalized for COVID, or dying of COVID) and level of social distancing endorsed (using Bonferroni-corrected *p*-value threshold of 0.008), (2) Brief Cope Scale subscales (corrected *p* < 0.004), (3) COVID Wellbeing subscales, where participants endorsed their level of worry concerning seven COVID-related domains (corrected *p* < 0.007), and (4) whether they endorsed that their college progress or performance had been impacted by the COVID-19 pandemic (corrected *p* < 0.025).

In addition, Mann-Whitney tests (using packages “stats” and “rstatix” (Kassambara, 2021)were used to compare groups on pre-pandemic scales related to risk and resilience factors. Given the number of scales collected in this regard (19 variables collected across three pre-pandemic time points), GFA was conducted to identify latent factors. GFA was performed using the “optmThrGFA” package (Forthman and Yeh, 2021) which extends the GFA package developed by Leppäaho et al. (2017) by optimizing the parameters. The GFA method developed by Leppäaho et al. builds on previous group factor analysis by applying an advanced structural sparsity prior that does not assume the groups are independent, enabling the examination of variance within a set of variables, but also covariance between the sets (i.e., time points) (Klami et al., 2015). The optmThrGFA package runs the GFA multiple times in order to identify robust factors (factors that are replicated across repetitions of the GFA). We sought to identify factors accounting for at least 5% of model variance either overall or within a group of variables (i.e., at each time point). Our subsequent analyses examining potential pre-pandemic risk and resilience factors relating to group (“increased” versus “low stable”) aimed to focused on any GFA factors identified as well as any individual variables of interest that did not load strongly onto a factor but were of specific interest in relation to outcomes (using Bonferroni correction for multiple comparisons).

If any set of the above Mann-Whitney or chi-squared analyses identified variables that may be meaningful in predicting group, we entered these variables into a stepwise binomial logistic regression using the “stats” (R Core Team, 2021) and “aod” (Lesnoff and Lancelot, 2012) packages to identify the combined utility of these variables for group prediction.

## Results

### Changes in symptoms over time

**FIGURE 1:**
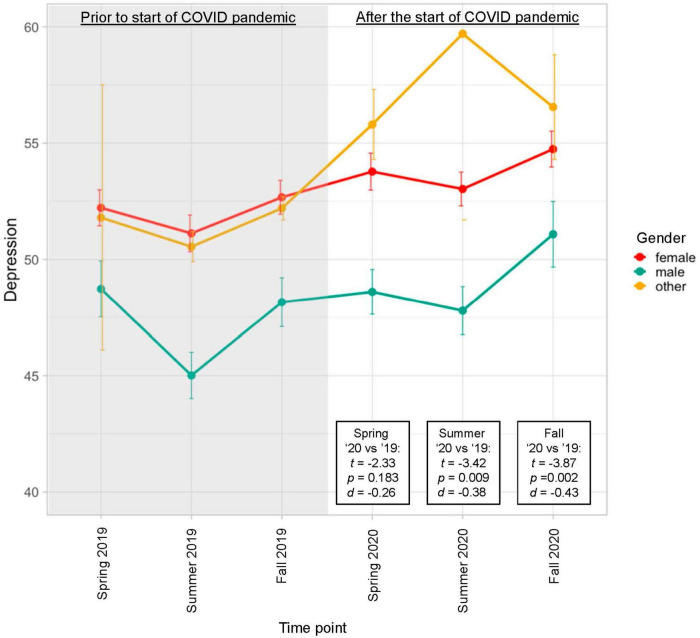
Average depression symptom severity reported over time by group. Linear mixed effects models (with gender included as a covariate, determined via BIC) revealed a significant increase in symptoms over time [*F*(5,807) = 10.36, *p* < 0.001]. T statistics listed in the figure were obtained from Tukey’s HSD tests comparing corresponding time points from 2020 to 2019.

Baseline demographics for the entire sample are shown in Table 1. Results from LMM (with gender included as a covariate, determined via BIC) and Tukey’s HSD tests are provided in Table 2. Results revealed a significant increase in depression symptoms over time (see Figure 1) and Tukey’s HSD tests indicated significantly greater depression symptoms for Summer 2020 compared with Summer 2019 and for Fall, 2020 compared with Fall, 2019, but not when comparing Spring 2020 with Spring 2019. The gender effect was characterized by higher depression symptoms reported by female than male participants. There was also an overall effect of increasing anxiety symptoms over time, but without significant differences when comparing the specific corresponding time points using Tukey’s HSD. The gender effect was again characterized by higher anxiety symptoms reported by female than male participants.

**TABLE 1:** Demographics.

	*N* = 183
Age, Mean (SD)	20.08 (1.33)
**Gender, N (%)**	
Female	123 (67.2%)
Male	58 (31.7%)
Other	2 (1.1%)
Ethnicity, *N* = Non-Hispanic (%)	166 (90.7%)
**Race, N (%)**	
American Indian	3 (1.6%)
Asian Indian	3 (1.6%)
Black	11 (6.0%)
Chinese	4 (2.2)%
Korean	2 (1.1%)
Middle Eastern	1 (0.5%)
Multi-Race	17 (9.3%)
Other	2 (1.1%)
Other Asian	5 (2.7%)
White	135 (73.8%)
**Annual parent or household income, N (%)**	
50,000 and less	59 (32.2%)
50,000 – $100,000	54 (29.5%)
100,000 – $150,000	34 (18.6%)
150,000 and over	36 (19.7%)
Psychotropic medication, *N* = yes (%)	11 (6.0%)
**Consent Year, N (%)**	
2016	45 (24.6%)
2017	63 (34.4%)
2018	75 (41.0%)
Resilience training, N (%)	80 (43.7%)
**College, N (%)**	
A&S college	48 (26.2%)
Business college	24 (13.1%)
Eng&NS college	67 (36.6%)
HS college	44 (24.0%)
**First in college, N (%)**	
Yes	24 (13.1%)
No	157 (85.8%)
Uncertain	2 (1.1%)
**Number completing each time point (based on PROMIS Depression scale)**	
Spring, 2019	171
Summer, 2019	153
Fall, 2019	176
Spring, 2020	151
Summer, 2020	183
Fall, 2020	155

A&S, arts and sciences; HS, health sciences; Eng&NS, engineering and natural sciences; PROMIS, patient reported outcome measurement information system.

**TABLE 2:** Changes in symptoms over time.

Variables		*F*	*p*	*t*	*p*	*Cohen’s d*	95% CI	
							Lower	Upper
Model for PROMIS depression		9.71	<0.001					
**Time**								
	Summer 2019 vs. 2020			−2.03	0.015	−0.36	−0.58	−0.14
	Fall 2019 vs. 2020			−2.40	0.002	−0.42	−0.64	−0.20
	Spring 2019 vs. 2020			−2.31	0.191	−0.26	−0.48	−0.038
**Gender**								
	Female vs. Male			10.43	<0.001	0.83	0.47	1.19
Model for PROMIS anxiety		9.42	<0.001					
**Time**								
	Summer 2019 vs. 2020			−1.80	0.102	−0.29	−0.50	−0.067
	Fall 2019 vs. 2020			−1.62	0.702	−0.26	−0.48	−0.037
	Spring 2019 vs. 2020			−1.74	0.508	−0.20	−0.42	−0.027
**Gender**								
	Female vs. Male			6.00	<0.001	0.95	0.57	1.33

PROMIS, patient reporting outcome measurement information system; CI, confidence interval. Degrees of freedom (df) for the time effect on depression and anxiety symptoms from the linear mixed model were df1 = 5, df2 = 799; the df = 799 for Tukey’s HSD (honestly significant difference) test comparing specific time points.

Subgroups were identified that exhibited “increased depression” (*N* = 63; an increase in >3.5 T points from the average of symptoms pre-pandemic to the average of symptoms since the start of the pandemic); “low stable depression” (*N* = 79; mean pre-pandemic symptoms < 55 and change of < 3.5 T-score points from before to after the start of the pandemic); and “high stable” depression (*N* = 41; mean pre-pandemic symptoms >55 and change of < 3.5 T-score points). Demographic information for these subgroups is provided in Supplementary Table 1. Notably, this original “increased” depression group was more female, Hispanic, and more likely to report household income < $100,000, corroborating how the pandemic may have disproportionately impacted some demographic groups more than others (Phiri et al., 2021). To support analyses examining psychological and COVID-related variables that may have predicted mental health trajectories, propensity matching was used to match the “low stable” group to the “increased” group in relation to gender, race (binary variable: minority, white), ethnicity (binary: Hispanic vs. non-Hispanic), cohort (2016, 2017, 2018), and pre-pandemic average PROMIS Depression score, resulting in groups of *N* = 63 each. See Table 3 for demographic information for these matched groups.

**TABLE 3:** Overall demographics with matched groups.

	Increased	Low stable	*p*
	(*N* = 63)	(*N* = 63)	
Age, Mean (SD)	19.97 (0.95)	19.91 (0.98)	0.75
**Gender, N (%)**			0.245
Female	46 (73.0%)	43 (68.3%)	
Male	15 (23.8%)	20 (31.7%)	
Other	2 (3.2%)	0 (0.0%)	
Ethnicity, *N* = Non-Hispanic (%)	52 (82.5%)	59 (93.7%)	0.099
**Race, N (%)**			0.327*
American Indian	0 (0.0%)	2 (3.2%)	
Asian Indian	2 (3.2%)	0 (0.0%)	
Black	2 (3.2%)	4 (6.3%)	
Chinese	1 (1.6%)	1 (1.6%)	
Middle Eastern	0 (0.0%)	1 (1.6%)	
Multi-Race	2 (3.2%)	5 (7.9%)	
Other	2 (3.2%)	0 (0.0%)	
Other Asian	1 (1.6%)	2 (3.2%)	
White	53 (84.1%)	48 (76.2%)	
Race, *N* = White (%)	53 (84.1%)	48 (76.2%)	0.372
**Annual parent or household income, N (%)**			0.222*
50,000 and less	21 (33.3%)	22 (34.8%)	
50,000 – $100,000	24 (38.1%)	13 (20.6%)	
100,000 – $150,000	8 (12.7%)	9 (14.3%)	
150,000 and over	10 (15.8%)	19 (30.1%)	
Parent income, *N* = less than $100,000 (%)	45 (71.4%)	35 (55.6%)	0.096
Financial Aid amount, Mean (SD)	24097.41 (14749.40)	27919.21 (15228.26)	0.176
**Psychotropic medication use, N (%)**			0.273*
Yes	1 (1.6%)	3 (4.8%)	
No	60 (95.2%)	58 (92.1%)	
NA	2 (3.2%)	2 (3.2%)	
**Therapy in past 3 months, N (%)**			1.00*
Yes	3 (4.8%)	3 (4.8%)	
No	58 (92.1%)	58 (92.1%)	
NA	2 (3.2%)	2 (3.2%)	
Past Therapy, *N* = no (%)	61 (96.8%)	61 (96.8%)	1.00*
**Consent Year, N (%)**			0.834
2016	17 (27.0%)	15 (23.8%)	
2017	19 (30.2%)	22 (34.9%)	
2018	27 (42.9%)	26 (41.3%)	
Resilience training, *N* = yes (%)	30 (47.6%)	30 (47.6%)	1.00*
**College, N (%)**			0.222
A&S college	20 (31.7%)	12 (19.0%)	
Business college	4 (6.3%)	11 (17.5%)	
Eng&NS college	21 (33.3%)	25 (39.7%)	
HS college	18 (28.6%)	15 (23.8%)	
**First in college, N (%)**			0.193*
Yes	11 (17.5%)	7 (11.1%)	
No	50 (79.4%)	56 (88.9%)	
Uncertain	2 (3.2%)	0 (0.0%)	

A&S, arts and sciences; HS, health sciences; Eng&NS, engineering and natural sciences. Independent samples t-tests were utilized to compare groups on continuous variables. Chi-square tests were used for testing differences group differences in categorical variables, except for those denoted with *, for which Fisher’s Exact tests were utilized due to small sample sizes in some cells.

### Group factor analysis of pre-pandemic risk/resilience factors

Only one factor explained >5% variance across blocks (Factor 1). This factor, exhibited moderate to high positive loadings for risk variables (i.e., >0.40 for sleep impairment, sleep disturbance, social isolation) and negative loadings for resilience variables (i.e., <−0.40 for emotional and information support, meaning and purpose, friendship, self-efficacy, and ERQ reappraisal; see Supplementary Figures 2, 3 and Supplementary Table 2 for further detail on factor analysis results and loadings). Thus, this factor was termed a “general risk factor.” No additional factors were identified that explained significant variance or included substantial loadings by more than one variable. Thus, in subsequent analysis to identify pre-pandemic risk/resilience variables that may predict trajectory of response to COVID, we used the Factor 1 score and the individual scores from the additional measures that did not load >0.30 onto this factor (i.e., alcohol use, cannabis use, and total occurrence of traumatic events; corrected *p* < 0.008).

### Variables collected after the start of the pandemic relating to symptom trajectory

Very few students in the current sample endorsed being diagnosed with COVID-19 (3 in the “increased depression,” 2 in the “low stable” depression groups) and there were no participants who endorsed that they had been hospitalized with COVID-19. The matched “increased” and “low stable” depression groups did not differ significantly on whether they knew someone who had COVID-19 (43 in “low stable” group; 40 in the “increased” group; *X*^2^ (1) = 1.00, *p* = 0.317, 1.00, *OR* = 1.40 [95% *CI*: 0.72 – 2.72]), had someone in the household who had COVID-19 (7 in “low stable” group; 6 in “increased” group; *X*^2^ (1) = 0.00, *p* = 1.00, *OR* = NA), or had a friend diagnosed with COVID-19 (8 in the “low stable” group; 7 in the “increased” group): *X*^2^ (1) = 0.34, *p* = 0.56, *OR* = 1.22 [95% *CI*: 0.63 – 2.38]). While those in the “increased” group reported knowing more people who had been hospitalized (*N* = 16 or 25%) or died (*N* = 7 or 11%) due to COVID-19 than those in the “low stable” group (hospitalized: *N* = 10 or 16%; death by COVID-19: *N* = 2 or 3%), though these differences were not statistically significant (hospitalized: *X*^2^ (1) = 1.21, *p* = 0.271, *OR* = 1.43 [95% *CI*: 0.76 – 2.70]; death by COVID-19: *X*^2^ (1) = 1.92, *p* = 0.167, *OR* = 1.43 [95% *CI* = 0.76 – 2.70]) though the ability to detect statistical differences was likely impacted by the low incidence rate. There were no group differences in the level of social distancing endorsed at either post-pandemic time point (Summer 2020: *W* = 2,043, *p* = 0.77, *r* = 0.026; Fall 2020: *W* = 1,332, *p* = 0.635, *r* = 0.046). There were group differences in political leanings, with the “increased” group rating themselves as somewhat more liberal on average (Summer: *W* = 1552.5, *p* = 0.033 *r* = 0.19; Fall: *W* = 1,073, *p* = 0.048, *r* = 0.19).

**FIGURE 2:**
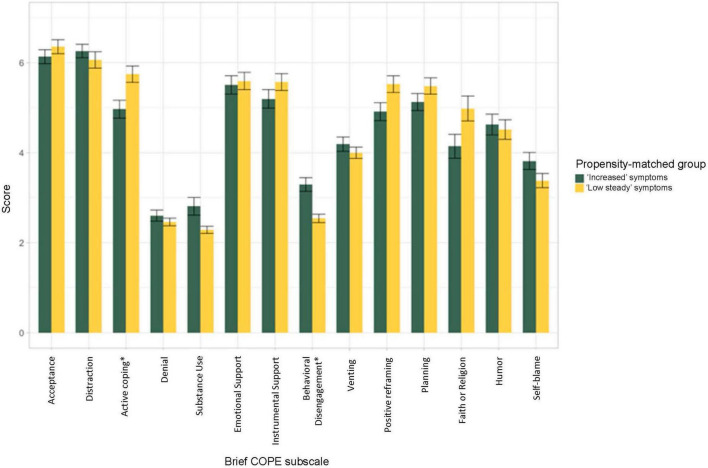
Average subscale score on the Brief Cope Scale. Subscale scores were averaged across Spring, Summer, and Fall 2020 time points. As compared with the “increased” depression group, the “low stable” depression group exhibited greater scores on the active coping (*W* = 1,380, *p* = 0.003, *r* = 0.26), positive reframing (*W* = 1,528, *p* = 0.026, *r* = 0.35), and religion subscales (*W* = 1506.5, *p* = 0.018, *r* = 0.21), as well as lower scores on the behavioral disengagement subscale (*W* = 2,752, *p* < 0.001, *r* = 0.35) of the Brief Cope scale (see Supplementary Table 3 for statistical results for all subscales), though only the active coping and behavioral disengagement subscales would meet specified multiple comparison correction thresholds (as indicated by “*”).

For the Brief Cope Scale, the “low stable” depression group (compared with the “increased” depression group) exhibited greater scores on the active coping (*W* = 1,380, *p* = 0.003, *r* = 0.26), positive reframing (*W* = 1,528, *p* = 0.026, *r* = 0.35), and religion subscales (*W* = 1506.5, *p* = 0.018, *r* = 0.21), as well as lower scores on the behavioral disengagement subscale (*W* = 2,752, *p* < 0.001, *r* = 0.35; see Figure 2 and Supplementary Table 3 for statistical results for all subscales), though only the active coping and behavioral disengagement subscales would meet specified multiple comparison correction thresholds. These four variables were entered into a stepwise binomial logistic regression predicting group status, which identified a model that included only active coping (*B* = −0.22, SE *B* = 0.13, *Z* = −1.67, *p* = 0.095; *OR*: 0.80 [95% *CI*: 0.62 – 1.04]) and behavioral disengagement (*B* = 0.71, SE *B* = 0.22, *Z* = 3.24, *p* = 0.001; *OR* = 2.03 [95% *CI*: 1.35 – 3.20]) and had a classification accuracy of 62.70%.

**FIGURE 3:**
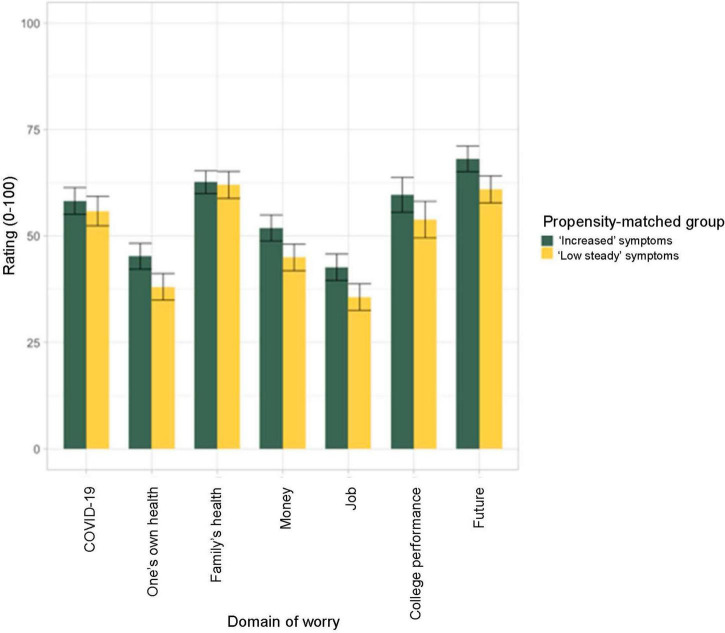
Average level of worry endorsed for different domains of concern related to the COVID-19 pandemic. Ratings were averaged across Spring, Summer, and Fall 2020 time points. Student on average reported worrying the most about their family’s health and their future. There were no significant differences between the propensity matched groups exhibiting “increased” depression symptoms or “low stable” depression symptoms from before to after the start of the COVID-19 pandemic.

Across groups, the areas that participants endorsed worrying about the most since the start of the pandemic was their family’s health and their future (see Figure 3). The “increased” and “low stable” depression groups did not differ significantly on any of the domains of worry associated with COVID-19 (i.e., about COVID, their own or family’s health, money, job, future, college performance; all *p*s > 0.10; see Supplementary Table 4 for full statistical results). However, those in the “increased” group were more likely to report their college progress being slowed due to COVID-19 then the “low stable” group (*X*^2^ (1) = 5.45, *p* = 0.020. *OR* = 2.16 [95% *CI*: 1.15 – 4.76]) but were not significantly more likely to endorse that their college performance had been impacted (endorsed by 19 in the “increased” group, 15 in the “low stable” group; *X*^2^ (1) = 0.31, *p* = 0.577 *OR* = 1.20 [95% *CI*: 0.64 – 2.25]).

### Variables collected pre-pandemic relating to symptom trajectory

In regard to pre-pandemic risk and resilience factors, the groups did not differ on the general risk factor identified by the GFA (*W* = 1,208, *p* = 0.908, *r* = 0.012) or on the total occurrence of past traumatic events (*W* = 2,096, *p* = 0.57, *r* = 0.048). However, there were trend differences in alcohol (*W* = 2,394, *p* = 0.038, *r* = 0.19) and cannabis use (*W* = 2,209, *p* = 0.092, *r* = 0.15), in which the “increased” depression groups exhibited higher levels of use pre-pandemic then the “low stable” group (see Supplementary Table 5 for descriptive and full statistical results). When entered into a stepwise binomial logistic regression, the model identified only included alcohol use (*B* = 0.14, SE *B* = 0.062, *Z* = 2.26, *p* = 0.024, *OR* = 1.15 [95% *CI*: 1.03 – 1.31]; 58% classification accuracy).

## Discussion

The current study examined (1) the trajectory of symptoms of depression and anxiety for a cohort of college students who were followed 1 year pre- to approximately 6 months post-onset of the COVID-19 pandemic, and (2) factors that may account for differentiation of the trajectories based on groups propensity-matched on pre-pandemic depression and sociodemographic factors. Results corroborate other reports indicating that students experienced worsening of mental health symptoms with the pandemic, with symptoms getting worse as the pandemic progressed and classes resumed in Fall 2020. Students who reported using more active coping strategies were less likely to exhibit worsening of symptoms with the pandemic. Similarly, those who were using more alcohol prior to the pandemic were more likely to experience worsening of symptoms. There was further indication that students with worsening mental health symptoms were also more likely to report their college progress being slowed, liberal political leanings, and have had someone in their household hospitalized or die due to COVID-19.

Although some studies have reported worsening mental health in the general population as a results of the COVID-19 pandemic, there have also been studies reporting a lack of change (Pirkis et al., 2021). Current results support prior studies indicating that the pandemic has a negative impact on mental health, particularly among younger adults (Son et al., 2020). Given the stressors of college (i.e., transition period, financial stress, clinically significant mental health symptoms, changing social networks, etc.) and increased mental health risk for this population more generally, it is perhaps understandable that college students may be vulnerable when there is the added stress of a negative world event such as the COVID-19 pandemic. Given the low rates of diagnosis and hospitalization due to COVID-19 in the current sample, it is difficult to conclude the mental health impact arising directly from COVID-19 infections (of oneself and family/friends); however, results corroborate how life stressors relating to the pandemic, which are likely influenced by numerous contextual and individual factors (e.g., financial resources, coping mechanisms, etc.), have a negative impact on college student mental health.

Also consistent with previous reports were findings that in the unmatched groups, those showing “increased” depression symptoms were more likely to be female, from under-represented race/ethnicity, and have lower income than those showing “low stable” depression symptoms. It has been suggested that some of these negative impacts may be due to the impact of the pandemic on jobs often occupied by women (e.g., retail, service industry, healthcare) and due to minority groups and those with lower income being hit the hardest by the COVID-19 virus and related economic impacts (Gonzales et al., 2020; Xiong et al., 2020; Charles et al., 2021). These factors may play a role directly for college students or by impacting their family support system. However, it is important to note that in our analysis of the matched groups, students with worsening symptoms reported, on average, more liberal political leanings. Overlayed onto the timeline of the COVID-19 pandemic was a tumultuous political climate in the United States, particularly concerning issues of immigration, gender, and race (Alang et al., 2020). It is possible that the worsening mental health observed during this time period may be due to a combination of factors, including not only direct impact of the COVID-19 pandemic, but also from political and governmental mistrust or racial unrest, which may have been experienced differently by individuals from various racial or ethnic backgrounds or by those with different political leanings.

Given that college occurs at an age in which the focus is on increasing independence and changing/increasing social networks, we expected the level of social distancing to be a factor contributing to the different trajectories of mental health. However, this hypothesis was not supported in the current data. Instead, it seemed that across the sample, the greatest source of worry was about their family’s health and their future. Students exhibiting a worsening trajectory of mental health tended to have more experiences with serious COVID-19 related illness in their family and were more likely to report their college progress being impacted. Thus, at least in this sample, academic and health-related concerns related to trajectory of mental health for college students more so than social distancing behavior. In addition to recognizing the external and societal factors contributing to mental health during the pandemic, it is also important to delve into individuals’ coping strategies that may provide protective effects against poor mental health outcomes. The current results suggest that active coping, or the process of taking active steps to try to remove or circumvent the stressor or to ameliorate its effects, may be one important resilience factor (Carver et al., 1989; Agha, 2021). Thus, while a pandemic may seem to be a negative life event in which the individual has very little control, active coping strategies may support identifying the aspects that are in their control and taking action to address those specific stressors. Maladaptive coping strategies on the other hand, such as behavioral disengagement and substance use, may serve as important risk factors among young adults (Czeisler et al., 2020; Horigian et al., 2020). Results suggest that engaging in heavier alcohol use during college may have deleterious effects on one’s ability to build resilience skills to optimally respond to future stressors.

Colleges should consider the strain that COVID-19 places on their students when crafting college-based policies. Given that active-based coping strategies may serve as a resilience factor against poor mental health outcomes; colleges should look to increase access to potentially beneficial coping strategies, including social support, such as mental health resources and group-based extracurricular organizations (Wang S. et al., 2020; Xiong et al., 2020; Cohen et al., 2021; Soga et al., 2021). Additionally, it is essential that colleges look to provide additional support, both financial and social, to at-risk groups to help support their academic success.

## Limitations

The students in this sample were those enrolled in a study examining clinical outcomes of a resilience-based intervention implemented during the first semester of college (Akeman et al., 2019). Unfortunately, students who completed the intervention did not seem to exhibit greater protection from the impact of the pandemic on mental health. While the intervention did not seem to have an impact, it is possible that generalizability to other samples may be limited by the fact that the current sample was from an intervention study. In addition, the sample size was based on power calculations for the original purpose of the study rather than for the current analyses. Thus, it is possible that some of the current analyses may have been underpowered. We also recognize that current findings relate to responses within approximately 6 months after the start of the COVID-19 pandemic and that further studies are needed to explore the longer-term mental health impact of the pandemic on college students.

## Conclusion

Researchers have long called for an increase in screening, programming, and accessible services to address the notable rise in college student mental health difficulties. As the COVID-19 pandemic has had, and continues to cause, a significant impact on the mental health, education, and daily routine of college students, it is more urgent than ever to evaluate and implement programming to address the needs of college students today. While the availability of the COVID-19 vaccines has dramatically decreased transmission rates and may support at least a partial return to “college as usual,” there are likely to be residual effects of the pandemic. This could include lasting mental health effects for subgroups of students, difficulties “catching up” to the prior expectations concerning academic progress and attainment, and potentially lasting negative impacts on the type and availability of job opportunities after graduation. It is prudent for universities and colleges to implement widespread programming focused on increasing resilience to stress and adversity through the use of active coping strategies; providing additional support as needed to women, lower income, and under-represented minority students; and to help support students’ academic progress despite the additional obstacles of the pandemic.

## Data availability statement

The raw data supporting the conclusions of this article will be made available by the authors, without undue reservation.

## Ethics statement

The studies involving human participants were reviewed and approved by the Western Institutional Review Board^®^ (WIRB^®^). The patients/participants provided their written informed consent to participate in this study.

## Author contributions

EA contributed to study design, data collection, intervention delivery, literature search, writing of the manuscript, and creation of figures and tables. MC contributed to the literature search, writing of the manuscript, and revisions to the manuscript. NK, KC, TM, DD, and EW contributed to data collection, intervention delivery, creation of figures, and revisions to the manuscript. ZC contributed to the creation of figures and revisions to the manuscript. KF contributed to data analyses and revisions to the manuscript. MP contributed to the study design and provided revisions to the manuscript. RA contributed to study design, data collection, supervision of intervention delivery, data analysis, literature search, and writing of the manuscript. All authors contributed to the article and approved the submitted version.
