# Supplementary material for: Active coping strategies and less pre-pandemic alcohol use relate to college student mental health during the COVID-19 pandemic
Source: Front Psychol. 2022 Aug 1;13:926697. doi: 10.3389/fpsyg.2022.926697 (PMC9376611; doi:10.3389/fpsyg.2022.926697)
Supplement: Supplementary file 1 [file Data_Sheet_1.docx]

| **Supplemental Table 1** | | | |
| --- | --- | --- | --- |
|  | Low Stable | Increased | *p* |
|  | (N = 79) | (N = 63) |  |
| Age, Mean (SD) | 20.11 (1.51) | 19.97 (0.95) | 0.537 |
| Gender, N (%) | |  | 0.012* |
| Female | 44 (55.7%) | 46 (73.0%) |  |
| Male | 35 (44.3%) | 15 (23.8%) |  |
| Other | 0 (0.0%) | 2 (3.2%) |  |
| Ethnicity, N = Non-Hispanic (%) | 75 (94.9%) | 52 (82.5%) | 0.035 |
| Race, N (%) |  |  | 0.179* |
| American Indian | 2 (2.5%) | 0 (0.0%) |  |
| Asian Indian | 1 (1.3%) | 2 (3.2%) |  |
| Black | 6 (7.6%) | 2 (3.2%) |  |
| Chinese | 3 (3.8%) | 1 (1.6%) |  |
| Middle Eastern | 1 (1.3%) | 0 (0.0%) |  |
| Multi-Race | 8 (10.1%) | 2 (3.2%) |  |
| Other | 0 (0.0%) | 2 (3.2) |  |
| Other Asian | 3 (3.8%) | 1 (1.6) |  |
| White | 55 (69.6%) | 53 (84.1%) |  |
| Race, N = White (%) | 55 (69.6%) | 53 (84.1%) | 0.07 |
| Annual parent or household income, N (%) |  |  | 0.266* |
| $50,000 and less | 24 (31.6%) | 21 (34.5%) |  |
| $50,000 - $100,000 | 17 (22.4%) | 23 (37.7%) |  |
| $100,000 - $150,000 | 14 (18.4% | 8 (13.1%) |  |
| $150,000 and over | 21 (27.7%) | 9 (14.8%) |  |
| Parent income, N = less than $100,000 (%) | 41 (53.9%) | 44 (72.1%) | 0.045 |
| Financial Aid amount, Mean (SD) | 27186.31 (15439.48) | 24097.41 (14749.40) | 0.254 |
| Psychotropic medication use, N = yes (%) | 3 (3.9%) | 1 (1.6%) | 0.629* |
| Therapy in past 3 months, N = yes (%) | 4 (5.3%) | 3 (4.9%) | 1.00* |
| Past Therapy , N = no (%) | 76 (100.0%) | 61 (100.0%) | NA |
| Consent Year, N (%) | |  | 0.442 |
| 2016 | 18 (22.8%) | 17 (27.0%) |  |
| 2017 | 32 (40.5%) | 19 (30.2%) |  |
| 2018 | 29 (36.7%) | 27 (42.9%) |  |
| Resilience Training, N = yes (%) | 36 (45.6%) | 30 (47.6%) | 0.941 |
| College, N (%) | | | 0.037* |
| A&S College | 13 (17.1%) | 19 (13.1%) |  |
| Business College | 16 (21.1%) | 4 (6.6%) |  |
| Eng&NS College | 30 (39.5%) | 21 (34.4%) |  |
| HS College | 17 (22.4%) | 17 (27.9%) |  |
| First in College, N (%) | | | 0.058* |
| Yes | 7 (9.2%) | 11 (18.0%) |  |
| No | 69 (90.8%) | 48 (78.7%) |  |
| Uncertain | 0 (0.0%) | 2 (3.3%) |  |
| Abbreviations: A&S, Arts and Sciences; HS, Health Sciences; Eng&NS, Engineering and Natural Sciences. Independent samples t-tests were utilized to compare groups on continuous variables. Chi-square tests were used for testing differences group differences in categorical variables, except for those denoted with a *, for which Fisher’s Exact tests were utilized due to small sample sizes in some cells. | | | |

| **Supplemental Table 2. Loadings for each variables across each block (time point) for Factor 1 identified via group factor analysis.** | | | | | | | | | | | |
| --- | --- | --- | --- | --- | --- | --- | --- | --- | --- | --- | --- |
|  |  |  | Fall (Block 3) | |  | Summer (Block 2) | |  | Spring (Block 1) | |  |
| Component | Block | Variable | Lower | Median | Upper | Lower | Median | Upper | Lower | Median | Upper |
| 1 | block_3 | Sleep Impairment | 0.279 | 0.461 | 0.612 | 0.311 | 0.475 | 0.663 | 0.276 | 0.439 | 0.612 |
| 1 | block_3 | Emotional Support | -0.751 | -0.606 | -0.416 | -0.679 | -0.505 | -0.35 | -0.729 | -0.573 | -0.431 |
| 1 | block_3 | Informational Support | -0.78 | -0.663 | -0.502 | -0.766 | -0.616 | -0.436 | -0.747 | -0.602 | -0.438 |
| 1 | block_3 | Sleep Disturbance | 0.312 | 0.471 | 0.626 | 0.246 | 0.449 | 0.599 | 0.29 | 0.453 | 0.62 |
| 1 | block_3 | Social Isolation | 0.501 | 0.644 | 0.759 | 0.433 | 0.581 | 0.736 | 0.415 | 0.572 | 0.714 |
| 1 | block_3 | Meaning and Purpose | -0.793 | -0.658 | -0.537 | -0.76 | -0.638 | -0.5 | -0.698 | -0.561 | -0.372 |
| 1 | block_3 | Friendship | -0.75 | -0.62 | -0.482 | -0.717 | -0.546 | -0.391 | -0.684 | -0.529 | -0.355 |
| 1 | block_3 | Self-Efficacy | -0.748 | -0.617 | -0.441 | -0.742 | -0.638 | -0.485 | -0.548 | -0.411 | -0.243 |
| 1 | block_3 | Perceived Stress | 0.191 | 0.322 | 0.474 | 0.051 | 0.218 | 0.363 | -0.008 | 0.169 | 0.334 |
| 1 | block_3 | CDRISC -10 Resilience | -0.786 | -0.651 | -0.501 | -0.845 | -0.711 | -0.575 | -0.816 | -0.674 | -0.513 |
| 1 | block_3 | Total trauma occurrence | -0.223 | -0.011 | 0.227 | -0.231 | -0.023 | 0.217 | -0.227 | 0.064 | 0.288 |
| 1 | block_3 | ERQ Reappraisal | -0.724 | -0.568 | -0.383 | -0.709 | -0.527 | -0.355 | -0.689 | -0.521 | -0.33 |
| 1 | block_3 | ERQ Suppression | -0.055 | 0.183 | 0.403 | -0.04 | 0.178 | 0.398 | -0.107 | 0.154 | 0.372 |
| 1 | block_3 | Epworth Sleepiness Scale | -0.028 | 0.223 | 0.414 | -0.058 | 0.183 | 0.377 | -0.049 | 0.179 | 0.384 |
| 1 | block_3 | Importance of Religion | -0.443 | -0.218 | -0.014 | -0.408 | -0.196 | 0.012 | -0.4 | -0.157 | 0.086 |
| 1 | block_3 | Alcohol Use | -0.006 | 0.208 | 0.396 | -0.026 | 0.202 | 0.422 | -0.037 | 0.174 | 0.363 |
| 1 | block_3 | Cannabis Use | -0.01 | 0.23 | 0.454 | 0.009 | 0.266 | 0.492 | 0.006 | 0.256 | 0.466 |
| 1 | block_3 | Amphetamine Use | -0.088 | -0.002 | 0.093 | -0.09 | -0.002 | 0.091 | -0.098 | 0.002 | 0.098 |
| 1 | block_3 | Opioid Use | -0.085 | 0.002 | 0.086 | -0.083 | -0.002 | 0.091 | -0.1 | 0.001 | 0.094 |
| Note: Patient-Reported Outcomes Measurement Information System (PROMIS): Sleep impairment, Emotional support, Informational support, Sleep disturbance, Social isolation, Meaning and purpose; National Institutes of Health Toolbox (NIH TB): Friendship, Self-Efficacy, and Perceived Stress; Connor-Davidson Resilience Scale (CDRISC-10); Traumatic Events Scale (TES): total trauma occurrence; Emotion Regulation Questionnaire (ERQ): reappraisal and suppression; Alcohol, Smoking, and Substance Involvement Screening Test (ASSIST): alcohol, cannabis, amphetamine, and opioid use. | | | | | | | | | | | |

| **Supplemental Table 3. Brief Cope Scale Results** | | | | | |
| --- | --- | --- | --- | --- | --- |
|  | Increased (N=63) | Low Stable  (N=63) |  | | |
| **Variable** | M (SD) | M (SD) | ***W*** | ***p*** | ***r*** |
| Acceptance | 6.13 (1.23) | 6.36 (1.24) | 1779 | 0.312 | 0.09 |
| Distract | 6.26 (1.18) | 6.06 (1.44) | 2079 | 0.642 | 0.042 |
| Active Coping | 4.97 (1.58) | 5.75 (1.44) | 1380 | 0.003 | 0.264 |
| Substance Use | 2.81 (1.56) | 2.29 (0.63) | 2229 | 0.118 | 0.139 |
| Denial | 2.60 (0.98) | 2.46 (0.67) | 2056 | 0.694 | 0.035 |
| Emotional Support | 5.51 (1.61) | 5.60 (1.52) | 1945.5 | 0.85 | 0.017 |
| Instrumental Support | 5.20 (1.63) | 5.57 (1.48) | 1747.5 | 0.246 | 0.104 |
| Behavioral Disengagement | 3.29 (1.20) | 2.54 (0.74) | 2752 | <.001 | 0.347 |
| Venting | 4.19 (1.27) | 4.00 (1.00) | 2125.5 | 0.488 | 0.062 |
| Positive Reframing | 4.91 (1.59) | 5.52 (1.47) | 1528.5 | 0.026 | 0.199 |
| Planning | 5.13 (1.50) | 5.48 (1.44) | 1721.5 | 0.198 | 0.115 |
| Religion | 4.14 (2.11) | 4.98 (2.20) | 1506.5 | 0.018 | 0.21 |
| Humor | 4.63 (1.83) | 4.52 (1.72) | 2052 | 0.743 | 0.03 |
| Self-blame | 3.82 (1.50) | 3.38 (1.25) | 2316 | 0.102 | 0.146 |

| **Supplemental Table 4. WELL Worries Scales** | | | | | |
| --- | --- | --- | --- | --- | --- |
|  | Increased (N=63) | Low Stable  (N=63) |  |  |  |
| **Variable** | M (SD) | M (SD) | ***W*** | ***p*** | ***r*** |
| COVID | 58.21 (24.84) | 55.83 (27.49) | 2092 | 0.602 | 0.047 |
| One's own health | 45.21 (24.15) | 38.05 (24.77) | 2322.5 | 0.100 | 0.147 |
| Family's health | 62.62 (21.15) | 61.96 (25.13) | 1923.5 | 0.768 | 0.027 |
| Money | 51.87 (24.26) | 44.94 (24.75) | 2307 | 0.116 | 0.14 |
| Job | 42.65 (24.66) | 35.63 (24.79) | 2317.5 | 0.105 | 0.145 |
| Future | 68.08 (23.99) | 60.91 (25.11) | 2338.5 | 0.085 | 0.154 |
| College performance | 59.64 (32.46) | 53.83 (34.07) | 1510.5 | 0.397 | 0.083 |

| **Supplemental Table 5. Pre-COVID Risk Factors** | | | | | |
| --- | --- | --- | --- | --- | --- |
|  | Increased  (N=63) | Low Steady  (N=63) |  | | |
|  | M (SD) | M (SD) | ***W*** | ***p*** | ***r*** |
| General Factor 1 | -0.22 (1.09) | -0.22 (0.85) | 1208 | 0.908 | 0.012 |
| Trauma total occurrence | 1.22 (1.66) | 1.00 (1.22) | 2096 | 0.567 | 0.048 |
| ASSIST Alcohol Use | 3.20 (3.97) | 1.79 (2.48) | 2394 | 0.038 | 0.185 |
| ASSIST Cannabis Use | 0.92 (2.32) | 0.43 (1.73) | 2209 | 0.092 | 0.15 |
| Importance of Religion | 4.47 (2.19) | 5.12 (1.86) | 1681.5 | 0.137 | 0.133 |
| PROMIS Emotional Support | 53.99 (8.46) | 54.64 (6.46) | 1988.5 | 0.986 | 0.002 |
| PROMIS Informational Support | 57.37 (8.19) | 57.66 (7.43) | 1996.5 | 0.955 | 0.005 |
| PROMIS Sleep Disturbance | 47.13 (7.44) | 45.82 (6.75) | 2265.5 | 0.171 | 0.122 |
| PROMIS Sleep Impairment | 50.55 (8.13) | 50.91 (7.29) | 1928.5 | 0.787 | 0.024 |
| PROMIS Social Isolation | 48.67 (8.32) | 48.48 (6.64) | 2081 | 0.64 | 0.042 |
| PROMIS Meaning and Purpose | 54.47 (9.56) | 56.12 (7.96) | 1794 | 0.353 | 0.083 |
| NIH TB Positive Affect | 49.97 (9.59) | 51.79 (7.66) | 1723.5 | 0.204 | 0.113 |
| NIH TB Friendship | 49.89 (9.76) | 52.44 (7.39) | 1727.5 | 0.211 | 0.112 |
| NIH TB Self-Efficacy | 47.86 (11.87) | 48.34 (10.78) | 1862.5 | 0.994 | 0.001 |
| NIH TB Perceived Stress | 59.05 (4.16) | 60.09 (4.35) | 1687 | 0.147 | 0.129 |
| CDRISC-10 | 29.98 (6.82) | 31.23 (5.65) | 1791 | 0.346 | 0.084 |
| ERQ Total | 4.55 (0.73) | 4.62 (0.81) | 1838 | 0.476 | 0.064 |
| ERQ Reappraisal | 5.20 (1.13) | 5.17 (1.11) | 2002.5 | 0.932 | 0.008 |
| ERQ Suppression | 3.58 (1.33) | 3.81 (1.11) | 1780.5 | 0.321 | 0.089 |
| ESS Total | 7.75 (3.97) | 7.46 (4.13) | 2075 | 0.66 | 0.039 |
| Abbreviations: ASSIST = Alcohol, Smoking, and Substance Involvement Screening Test; PROMIS = Patient-Reported Outcomes Measurement Information System; NIH TB = National Institutes of Health Toolbox; CDRISC-10 = Connor-Davidson Resilience Scale; ERQ = Emotion Regulation Questionnaire; ESS = Epworth Sleepiness Scale. | | | | | |

**Figure S1. COVID timeline.**

A timeline of state and local government restrictions and trajectory of total cases in the region in relation to study survey time points.

**Figure S2. Variance explained by factor.**

Variance explained by factor across and within each block (Spring, Summer and Fall 2019 time points) of variables associated with risk and resilience.

**Figure S3. Variable loadings.**

Pictorial representation of variable loadings for each of the 10 factors identified via the group factor analysis.
