# Supplementary figures and images for: Active coping strategies and less pre-pandemic alcohol use relate to college student mental health during the COVID-19 pandemic
Source: Front Psychol. 2022 Aug 1;13:926697. doi: 10.3389/fpsyg.2022.926697 (PMC9376611; doi:10.3389/fpsyg.2022.926697)

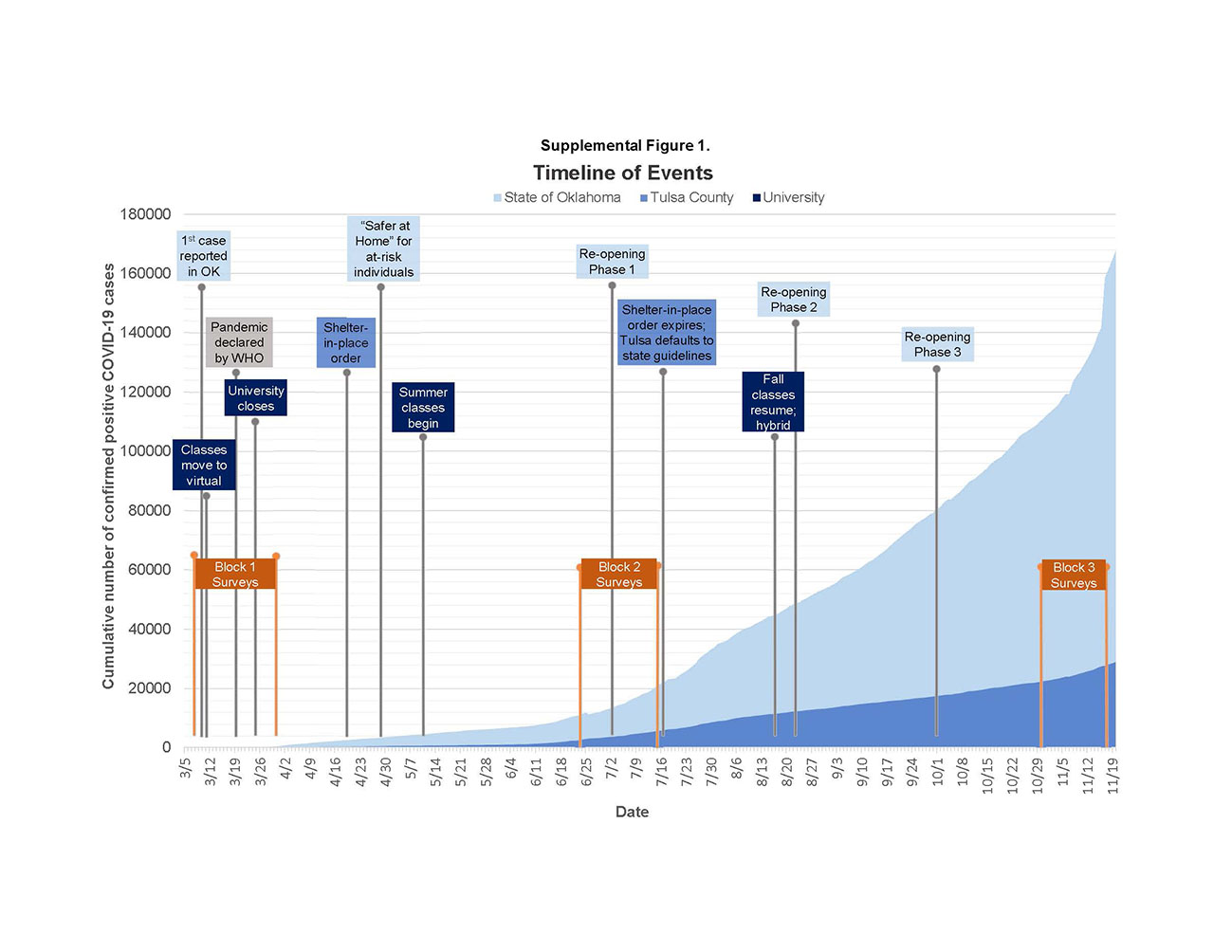

Supplement: Supplementary file 2 [file Image_1.JPEG]

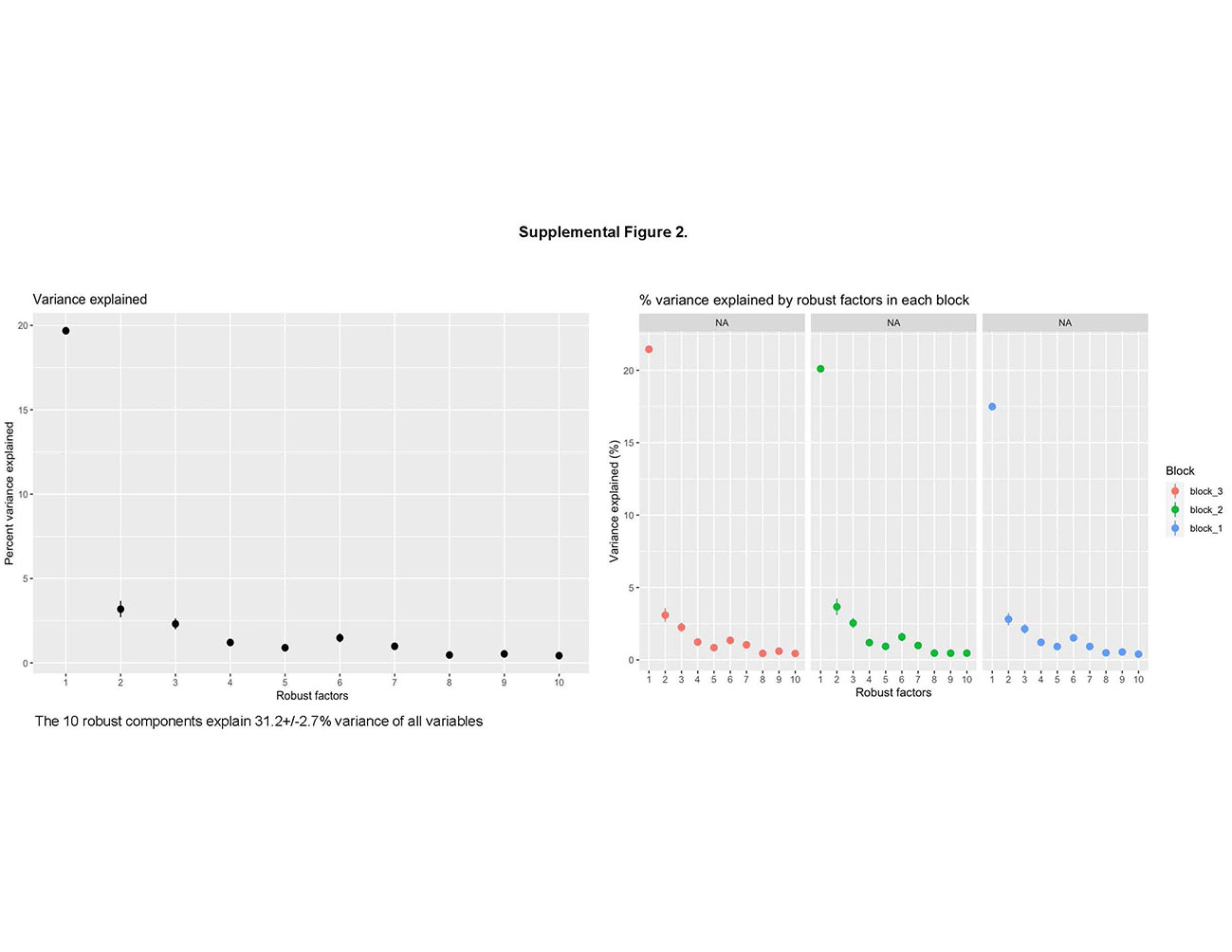

Supplement: Supplementary file 3 [file Image_2.JPEG]

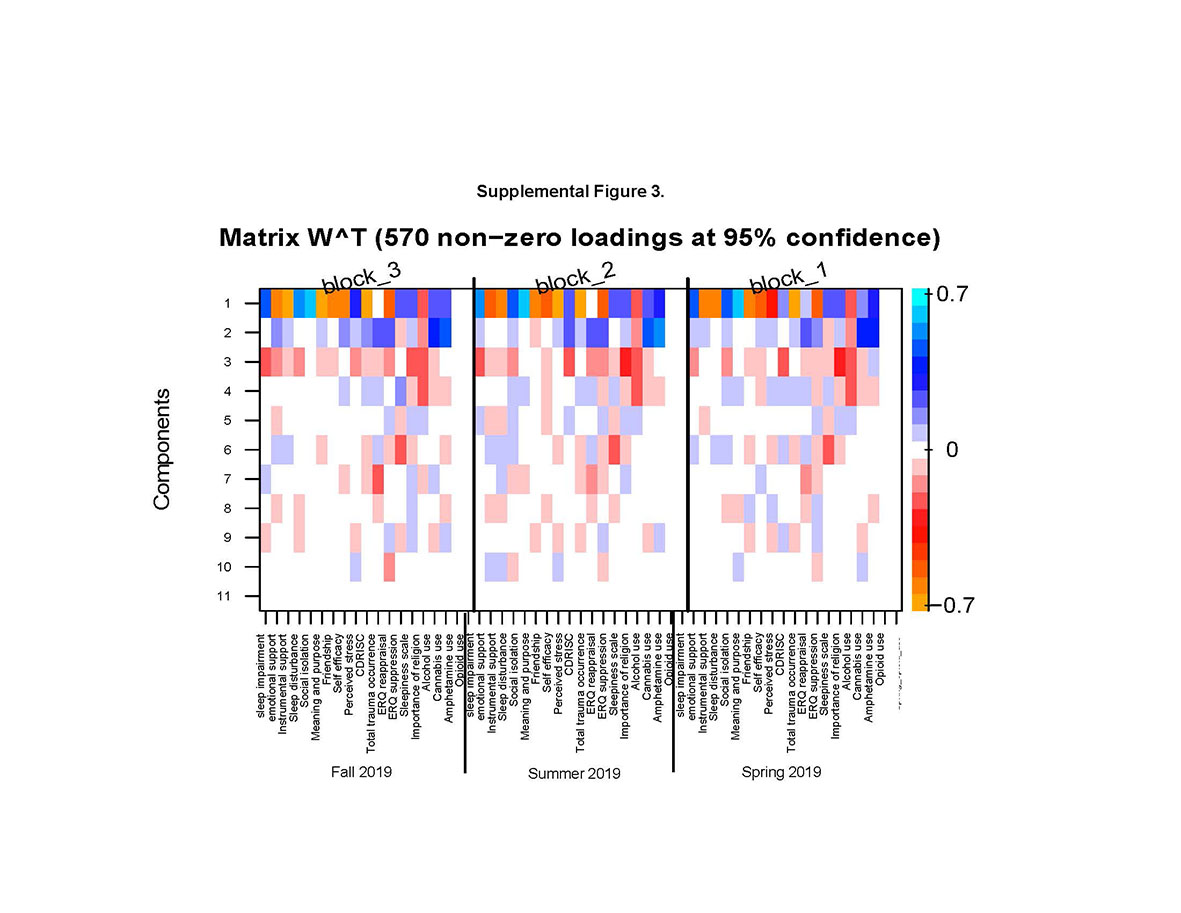

Supplement: Supplementary file 4 [file Image_3.JPEG]
